# Ascertainment of uninterrupted CAG repeat length and disease-modifying variants in fragment-based genetic testing for Huntington Disease

**DOI:** 10.1016/j.gimo.2024.101882

**Published:** 2024-08-02

**Authors:** Hailey Findlay Black, Chris Kay, Jessica Dawson, Stephanie Bortnick, Kyla Javier, Qingwen Xia, Cheuk Hin Chau, Tess Leavitt, Larissa Arning, Huu Phuc Nguyen, Michael R. Hayden

**Affiliations:** 1 Centre for Molecular Medicine and Therapeutics, University of British Columbia, Vancouver, BC, Canada; 2 Department of Human Genetics, Medical Faculty, Ruhr University of Bochum, Bochum, Germany

**Keywords:** Genetic modifiers, Huntington disease, Loss of interruption, Triplet-primed PCR, Variant screening

## Abstract

**Purpose:**

In Huntington disease (HD), synonymous variants causing loss or duplication of the interrupting CAA codon in the *HTT* CAG repeat modify disease onset. These variants are undetectable during HD genetic testing, resulting in inaccurate diagnostic reporting of uninterrupted CAG repeat length. Inaccurate reporting of CAG repeat length results in misdiagnosis of individuals with alleles near diagnostic cut-offs. We present a method to identify variant alleles during CAG repeat genotyping, allowing accurate diagnostic reporting of uninterrupted CAG repeat length.

**Methods:**

We used triplet-primed PCR (TP-PCR) to amplify *HTT* CAG repeat alleles with canonical or noncanonical repeat interruptions and leveraged differences in peak amplification patterns to develop a screening method based on peak height ratio (PHR). We used PHR to screen blood DNA from a cohort of symptomatic individuals with diagnostic CAG repeat lengths of 40 to 41.

**Results:**

TP-PCR enables accurate reporting of uninterrupted CAG repeat length in diagnostic testing by detecting HD alleles with loss or duplication of the CAG repeat interruption.

**Conclusion:**

PHR screening of TP-PCR traces is a cost-effective screening method for detection, ascertainment of uninterrupted *HTT* CAG repeat length, and accurate diagnostic reporting for individuals with disease-modifying noncanonical CAG repeat interruptions.

## Introduction

Huntington disease (HD, OMIM:143100) is a fatal autosomal-dominant neurodegenerative disorder caused by expansion of a polyglutamine-coding CAG repeat in the *Huntingtin* gene (*HTT*, HGNC:4851).^1^, ^2^, ^3^

Clinically, the longer CAG repeat allele determines an individual’s diagnostic category. Individuals with fully penetrant alleles (>39 CAG repeats) will develop HD within typical lifespans and those with reduced penetrance (RP) alleles (36-39 CAG repeats) may develop HD.^2^^,^^3^ Repeat lengths <36 are typically considered nonpathogenic under current diagnostic criteria.^2^, ^3^, ^4^, ^5^ Longer uninterrupted CAG repeat length is correlated with earlier age at motor symptom onset (AO).^6^^,^^7^

Most HD alleles share a canonical repeat sequence: a polyglutamine-coding CAG repeat interrupted by a penultimate synonymous CAA codon, followed by a shorter polyproline-coding CCG repeat interrupted by a synonymous CCA codon at the second position (CAG_n_-CAA-CAG-CCG-CCA-CCG_n,_ Supplemental Figure 1).^8^ However, HD also occurs on alleles with non-canonical repeat interruptions (Supplemental Figure 1), several of which are cis-acting AO modifiers.^8^, ^9^, ^10^, ^11^, ^12^, ^13^

The best-characterized noncanonical repeat variant (NCRV) is the loss of interruptions in both the CAG and CCG repeats (CAG-CCG LOI), associated with mean AO approximately 10 years earlier than expected based on uninterrupted CAG repeat length.^8^, ^9^, ^10^, ^11^ Loss of only the CCG repeat interruption (CCG LOI) is associated with similarly early onset.^13^ Loss of interruption in the CAG repeat alone (CAG LOI) occurs rarely, limiting statistical analysis, but also trends toward early onset.^12^ Conversely, duplication of the CAG repeat interruption (CAG DOI) is associated with later AO in some, but not all, cohorts.^8^^,^^10^^,^^12^

AO-modifying effects of NCRVs were not recognized until 2019,^8^, ^9^, ^10^ because these variants often go undetected in routine HD genetic testing, which measures CAG repeat length by fragment analysis. Typical assays amplify *HTT* CAG repeats by polymerase chain reaction (PCR) and size-separate PCR fragments using capillary electrophoresis.^2^ Because fragment analysis measures only amplicon length, not sequence composition, it cannot identify NCRVs.

Concerningly, diagnostic CAG repeat lengths determined by fragment analysis inaccurately report uninterrupted CAG repeat length for NCRVs with noncanonical CAG repeat interruptions, typically underestimating CAG-CCG LOI and CAG LOI alleles by 2 repeats, and overestimating CAG DOI alleles by 2 repeats.^8^, ^9^, ^10^, ^11^ Because uninterrupted repeat length best predicts AO,^8^^,^^9^^,^^11^ estimated AO for individuals with undetected NCRVs can be doubly inaccurate, because of combined mis-sizing and AO-modifying effects.

Moreover, inaccurate reporting of uninterrupted CAG repeat length in noncanonical *HTT* CAG repeat alleles may cause misdiagnosis, as demonstrated by a report of symptomatic HD in an individual in whom a CAG-CCG LOI allele with 36 uninterrupted CAG repeats was mis-sized as a nonpathogenic (CAG)34 allele.^5^ Similarly, at diagnostic CAG repeat lengths 38 to 39, where >15% of symptomatic individuals have CAG-CCG LOI variants, LOI variant alleles are erroneously reported as RP alleles (36-39 repeats) despite having FP uninterrupted repeat lengths (≥40 repeats).^11^

NCRV detection during genetic testing is critical for accurately reporting uninterrupted CAG repeat length. However, clinical NCRV screening has been impractical because previously used detection methods are inefficient or costly for large-scale use.^8^, ^9^, ^10^, ^11^, ^12^ To address this, we developed an easily implemented screen, based on widely used fragment sizing methods, to detect variants in the *HTT* CAG repeat and determine uninterrupted CAG repeat length in a clinical diagnostic setting.

Triplet-primed PCR (TP-PCR) is already used for HD testing by some clinical laboratories, successfully amplifies CAG, CCG, and CAG-CCG LOI variants, and detects CAA interruptions within the CAG repeat.^2^^,^^14^ Here, we demonstrate that TP-PCR differentiates CAG-CCG LOI, CAG LOI, and CAG DOI variants from canonical alleles based on differential primer binding, enabling detection of these disease-modifying variants and accurate reporting of their uninterrupted CAG repeat lengths.

## Methods

### *HTT* repeat sizing by standard and TP-PCR

We performed standard CAG repeat genotyping as described previously, using fluorescently labeled primers to amplify CAG, CCG, and CAG + CCG repeat regions to exclude allelic dropout (Supplemental Figure 2).^15^^,^^16^ We determined repeat lengths relative to DNA from individuals with known-length alleles, using the ABI 3500XL Genetic Analyzer and GeneMapper5 for analysis.

For TP-PCR sizing of CAG repeats, we used primers 5’-HEX-CCTTCGAGTCCCTCAAGTCCTTC-3’ (forward) and 5’-GTTTCGGCTGTTGCTGCTGCTGCTGCTG-3 (reverse), and sized CCG and CAG + CCG repeat regions as above. Cycle conditions were: 7 minutes denaturation (95 °C), 36 cycles (1 minute at 95 °C, 1 minute at 66 °C, and 1 minute at 72 °C), and 15 minutes extension (72 °C). We used Roche GMP-Grade Taq polymerase (03734927001) for all reactions and analyzed sizing results using GeneMapper5 software.

### Peak ratio calculation for variant screening

For NCRV screening, we calculated peak height ratios (PHRs) for upper alleles by dividing height of the main peak (CAG repeat length *n*) by the sum of peak heights at lengths *n* through *n +* 3. For alleles with low PHR relative to their apparent CAG length, we calculated secondary PHRs (2° PHRs) by dividing *n* + 2 peak height by the sum of *n* through *n +* 3 peak heights to differentiate LOI from DOI variants. We sequenced suspected variants as previously described.^9^ Each assay included known-length controls to enable between-batch PHR comparison, and we performed all statistical analyses in R (v4.0.4).

### Cohort information

For method development, we used sequence-confirmed canonical and NCRV blood DNA from 47 previously identified participants in the University of British Columbia (UBC) and Bochum HD Biobanks.^9^^,^^11^^,^^17^ To extend previous screening of symptomatic RP-range individuals,^11^ we assembled an unbiased screening cohort of all 122 unrelated symptomatic UBC HD Biobank participants with available blood DNA, known AO, and diagnostic CAG repeat lengths of 40 to 41.

## Results

### TP-PCR amplifies CAG-CCG LOI, CAG LOI, CCG LOI, and CAG DOI alleles

**Figure 1:**
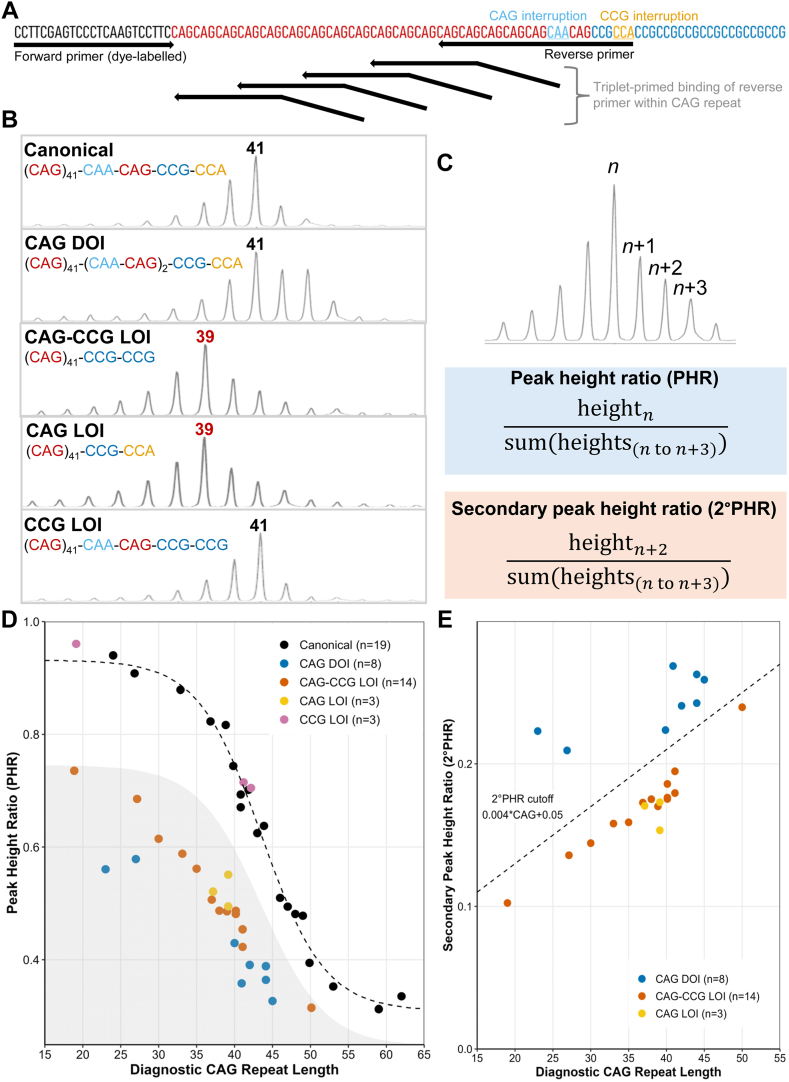
**Triplet-primed PCR and peak height ratio screening methods.** A. *HTT* canonical CAG and CCG repeat structure, indicating locations of interrupting codons and primer binding sites for triplet-primed PCR (TP-PCR) forward and reverse primers. B. TP-PCR traces of canonical, CAG duplication of interruption (DOI), CAG-CCG loss of interruption (LOI), CAG LOI, and CCG LOI alleles with uninterrupted CAG repeat lengths of 41; bold numbers show diagnostic CAG repeat lengths, and red numbers indicate misestimation of uninterrupted CAG repeat length. C. Peak height ratio (PHR) and secondary peak height ratio (2° PHR) calculations, with TP-PCR trace indicating peak naming notation. D. Effect of diagnostic CAG repeat length and genotype on PHR in TP-PCR sizing of blood DNA samples. Dashed line indicates logistic fit for canonical samples; grey shaded area indicates PHRs <80% of predicted value. E. 2° PHR enables CAG-length-dependent separation of DOI from LOI variants. Dashed line indicates proposed threshold for differentiation of LOI and DOI variants.

By TP-PCR (Figure 1A), we amplified blood DNA from individuals with identical uninterrupted CAG repeat lengths of 41 and known genotypes (canonical, CAG DOI, CAG-CCG LOI, CAG LOI, and CCG LOI). TP-PCR correctly sized canonical, CAG DOI, and CCG LOI alleles at 41 repeats but underestimated CAG-CCG LOI and CAG LOI alleles at 39 repeats (Figure 1B). These results are consistent with previous sizing by standard PCR, except for the CAG DOI variant, which is mis-sized at 43 repeats by standard PCR (Supplemental Figure 3).

Variants with noncanonical CAG repeat interruptions (CAG-DOI, CAG-CCG LOI, and CAG LOI) show distinct peak morphology, with peak heights to the right of the main peak (*n)* increased relative to the canonical allele. CAG-CCG LOI and CAG LOI variants have near-identical appearance, whereas a taller *n* + 2 peak distinguishes the CAG DOI variant. Differential primer binding at the interrupting region explains these peak morphology differences (Supplemental Figures 4-7). The CCG LOI variant is indistinguishable from canonical because the CAG repeat interruption is unchanged.

### PHR screening identifies alleles with noncanonical CAG repeat interruptions

Because noncanonical CAG repeat interruptions are distinguishable by TP-PCR, we established a PHR (Figure 1C) to identify them. To determine whether PHR is a useful screening tool, we performed TP-PCR and calculated PHRs for 47 known-genotype alleles.

In this data set, PHR decreases inversely to CAG repeat length determined by TP-PCR, and PHRs for CAG-CCG LOI, CAG LOI, and CAG DOI variants are distinguishable from canonical PHRs at each repeat length (Figure 1D). We fitted the relationship between PHR and repeat length in canonical alleles using a 4-parameter logistic model. The function $\text{PHR}=0.932+\frac{-0.622}{1+e^{\frac{43.548-\text{CAG}}{4.172}}}$ closely models canonical PHRs, and its asymptotes of 0.309 and 0.932 are consistent with the range of possible PHR values (0.25-1). Notably, PHRs for all CAG-CCG LOI, CAG LOI, and CAG DOI variants were <80% of predicted PHR for their diagnostic repeat lengths (Figure 1D, shaded region).

PHR cannot distinguish CAG-CCG LOI, CAG LOI, and CAG DOI variants. However, calculating a secondary PHR (2° PHR, Figure 1C) separates DOI variants from LOI variants (Figure 1E). In our data set, CAG-DOIs had 2° PHRs >0.004×CAG+0.05, whereas CAG-CCG and CAG LOIs fell below this threshold.

### PHR detects and distinguishes CAG-CCG LOI and CAG DOI variants among diagnostic (CAG)40-41 alleles

To test the efficacy of PHR screening for identifying noncanonical CAG repeat interruptions, we performed TP-PCR on DNA from an unbiased cohort of all 122 symptomatic probands with known AO, and diagnostic (fragment based) CAG repeat lengths of 40 (*n* = 42) and 41 (*n* = 80). This cohort included 3 known CAG-CCG LOI variants (underestimated as (CAG)40-41 alleles despite uninterrupted (CAG)42-43 repeat lengths), and 28 known canonical alleles based on prior clonal sequencing of the proband (*n* = 11) or an affected relative (*n* = 18). To assess PHR screening for DOI alleles, we added 2 known CAG DOI variants (excluded during cohort selection because of lack of AO data) with uninterrupted CAG repeat lengths of 40 and 41.

**Figure 2:**
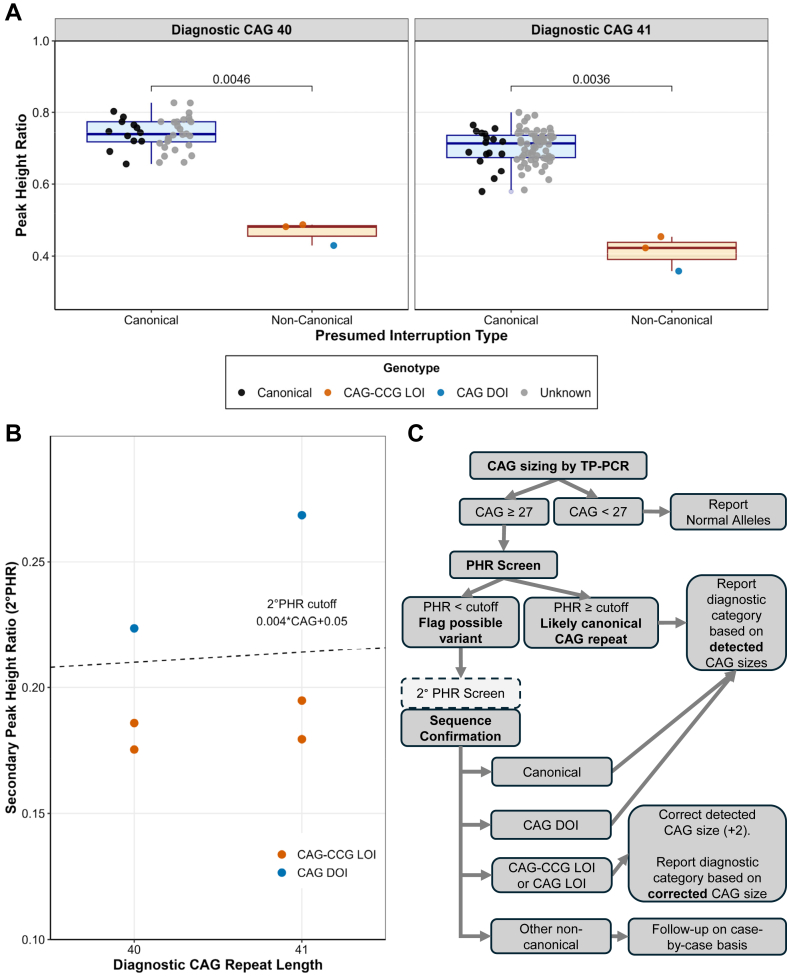
**Peak height ratio screening of (CAG)40-41 blood DNA.** A. Peak height ratio (PHR) values of (CAG)40-41 alleles with known canonical, known noncanonical, or unknown presumed-canonical genotypes. *P* values indicate results of Wilcoxon rank-sum tests. B. Secondary peak height ratio (2° PHR) values of (CAG)40-41 alleles with low PHR. Dashed line indicates the previous proposed threshold for differentiation of loss of interruption (LOI) from duplication of interruption (DOI) variants. C. Proposed screening workflow for identification and size-correction of mis-sized variant alleles, including optional 2° PHR screening of suspected variants.

We identified 3 (CAG)40 alleles and 3 (CAG)41 alleles with low PHRs (Figure 2A). Of these, 3 were known CAG-CCG LOI variants, 2 were known CAG DOI variants, and the last was identified by clonal sequencing as a CAG-CCG LOI variant. Therefore, we estimate CAG-CCG LOI variant frequencies in symptomatic HD at 4.8% and 2.5% for diagnostic CAG repeat lengths 40 and 41, respectively.

Because canonical and unknown-genotype individuals did not significantly differ in PHR (Supplemental Figure 8) or AO (Supplemental Figure 9), we combined them into a single group of presumed-canonical alleles. Among presumed-canonical alleles, mean PHR was 0.741 at (CAG)40, and 0.704 at (CAG)41, closely matching model predictions of 0.746 and 0.713. Among noncanonical individuals, mean PHRs at (CAG)40 and (CAG)41 were 0.466 and 0.412 respectively, and all PHRs fell below our previously established threshold (80% of predicted PHR). Next, we calculated 2° PHRs for these low-PHR alleles and found that our previously established threshold accurately separated CAG-CCG LOI from DOI variants (Figure 2B).

Based on pilot PHR screening of (CAG)40-41 individuals, we generated a proposed variant detection workflow (Figure 2C). Briefly, following TP-PCR sizing, alleles with ≥27 CAG repeats are PHR-screened, and below-threshold alleles are flagged as suspected variants. Low-PHR alleles undergo 2° PHR screening and confirmatory sequencing to identify LOI variants, for which detected repeat lengths are corrected (increased by 2 repeats) to accurately report uninterrupted CAG repeat length for CAG-CCG and CAG LOI variants. No repeat length correction is needed for CAG DOI variants or alleles with normal PHR.

## Discussion

TP-PCR is already the primary sizing method in some clinical laboratories because it improves large allele detection and reduces allelic dropout.^2^^,^^14^ The addition of PHR screening enables detection of CAG DOI, CAG-CCG LOI, and CAG LOI variants, providing an efficient variant identification method, requiring only data already generated by TP-PCR sizing.

Unlike standard CAG repeat amplification, TP-PCR accurately determines uninterrupted CAG repeat lengths of CAG DOI alleles and enables detection of inaccurately sized CAG-CCG LOI and CAG LOI variants by PHR screening. Although we recommend verifying all suspected variants by sequencing, 2° PHR allows provisional separation of LOI variants requiring CAG repeat length correction from accurately sized DOI alleles.

Identifying repeat sequence, not just fragment length, is critical for accurate HD testing, because it allows correct diagnostic categorization of noncanonical CAG repeat interruption variants. Accurate sizing is also valuable for clinical studies, which assume uninterrupted CAG repeat length and associated CAG-age-product scores^18^ during recruitment or analysis. Finally, increasing evidence indicates several NCRVs modify HD AO.^9^, ^10^, ^11^, ^12^ As functional consequences of these variants become better understood, variant identification may aid clinical trial stratification, and individuals undergoing genetic testing may wish to learn their variant status.^19^

Individuals at-risk for HD are often motivated to seek predictive testing by desire to plan their futures or make reproductive choices,^20^ but for those with undetected variants, conventional genetic testing misrepresents information necessary to make informed decisions. Individuals with reported (CAG)34 alleles expect not to develop HD and believe that their children’s risk of inheriting pathogenic expansions is low. However, those with CAG-CCG LOI variants have uninterrupted (CAG)36 repeat lengths, meaning that they and their children may develop HD.^5^ Similarly, individuals with reported (CAG)38 alleles might make career decisions believing they are unlikely to become symptomatic until late in life, if ever, but those with CAG-CCG LOI variants are not only expected to develop HD, given their FP uninterrupted (CAG)40 repeat lengths, but also to become symptomatic in their 40s.^11^ Moreover, we identified CAG-CCG LOI variants in 4.8% of symptomatic individuals with diagnostic (CAG)40, and 2.5% of those with diagnostic (CAG)41, indicating that even at common FP repeat lengths, notable numbers of individuals carry undetected and mis-sized onset-hastening variant alleles.

We expect that repeat sequencing, which most comprehensively detects known and novel variants, will eventually become standard practice for HD testing, but cost and technological barriers currently limit implementation. TP-PCR with PHR screening is a cost-effective alternative that enables detection and improved sizing of NCRVs with only minor changes to current testing protocols.

## Data Availability

Data will be made available on request (contact Dr. Michael Hayden, mrh@cmmt.ubc.ca).

## Conflict of Interest

Michael R. Hayden is the CEO of Prilenia Therapeutics, a private company, and serves on the public boards of Ionis Pharmaceuticals, Oxford Biomedica, AbCellera and 89bio. All other authors declare no conflicts of interest.
