# Supplementary material for: Ascertainment of uninterrupted CAG repeat length and disease-modifying variants in fragment-based genetic testing for Huntington Disease
Source: Genet Med Open. 2024 Aug 2;2:101882. doi: 10.1016/j.gimo.2024.101882 (PMC11613659; doi:10.1016/j.gimo.2024.101882)
Supplement: Supplementary Material [file mmc1.pdf]

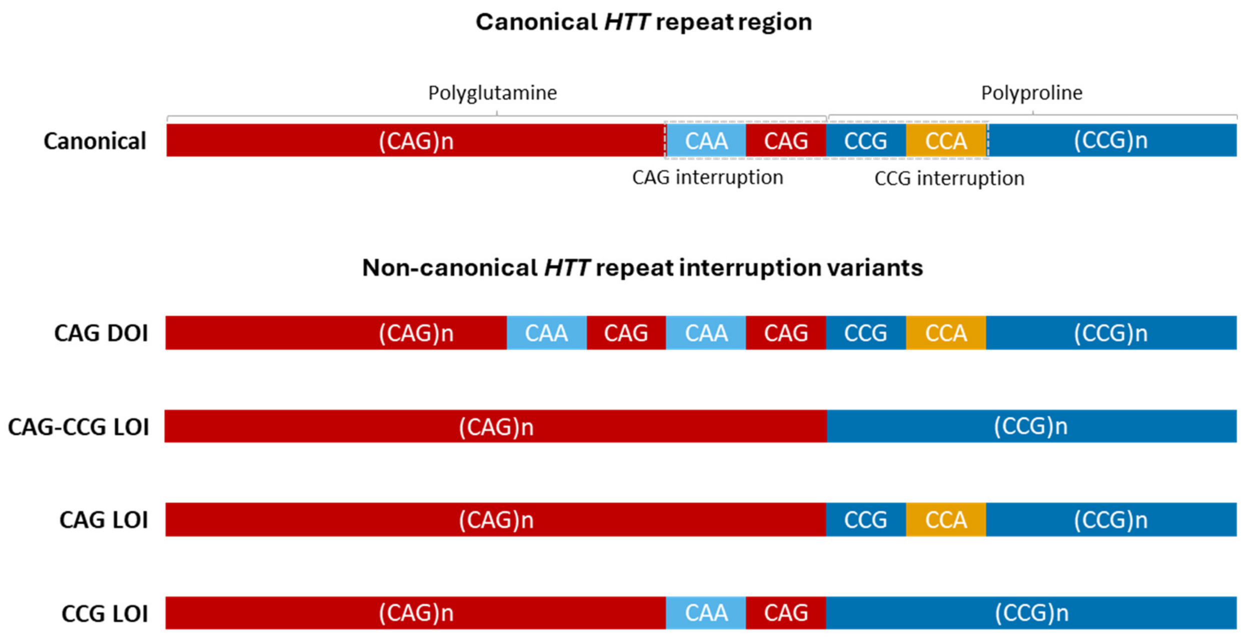


*Supplementary 1: Canonical and non-canonical HTT CAG and CCG repeat regions*

Diagram of the CAG and CCG repeat regions in *HTT* exon 1, indicating polyglutamine- and polyproline- coding sequences, as well as interrupting codons in the CAG and CCG repeats. Shown are the canonical sequence, as well as non-canonical repeat variants with duplication of interruption (DOI) of the CAG repeat (NM_002111.8:c.111G>A) or loss of interruption (LOI) of the CAG (NM_002111.8:c.117A>G) and/or CCG (NM_002111.8:c.126A>G) repeats.

| PCR Reaction | Forward Primer | Reverse Primer |
|---|---|---|
| CAG Repeat (Standard) | HD344F-HEX<br>5'-HEX-CCTTCGAGTCCCTCAAGTCCTTC | HD450R-PT<br>5'-GTTTGGCGGCGGTGGCGGCTGTTG |
| CCG Repeat (Standard) | HD419F-NED<br>5'-NED-AGCAGCAGCAGCAACAGCC | HD482R-PT<br>5'-GTTTGGCTGAGGAAGCTGAGGAG |
| CAG+CCG Repeat (Standard) | HD344F-FAM<br>5'-FAM-CCTTCGAGTCCCTCAAGTCCTTC | HD482R-PT<br>5'-GTTTGGCTGAGGAAGCTGAGGAG |
| CAG Repeat (Triplet-Primed) | HD344F-HEX<br>5'-HEX-CCTTCGAGTCCCTCAAGTCCTTC | HDTPR-PT<br>5'-GTTTCGGCTGTTGCTGCTGCTGCTGCTG |

*Supplementary 2: Primer sequences*

Table of primer sequences used for standard and triplet-primed polymerase chain reaction (PCR) amplification of the *HTT* CAG and/or CCG repeat regions for fragment analysis.

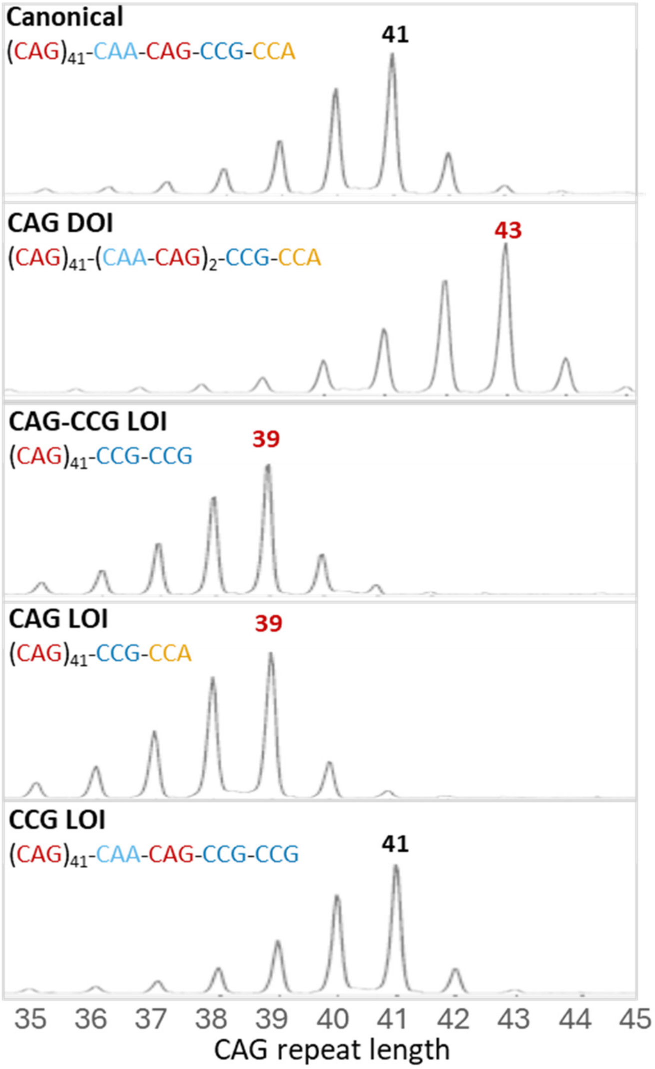


*Supplementary 3: CAG repeat amplification of canonical and variant alleles by standard PCR*

Standard PCR traces for CAG repeat amplification of canonical, CAG duplication of interruption (DOI), CAG-CCG loss of interruption (LOI), CAG LOI, and CCG LOI alleles with uninterrupted CAG repeat lengths of 41; bold numbers show diagnostic CAG repeat lengths, and red numbers indicate mis-estimation of uninterrupted CAG repeat length.

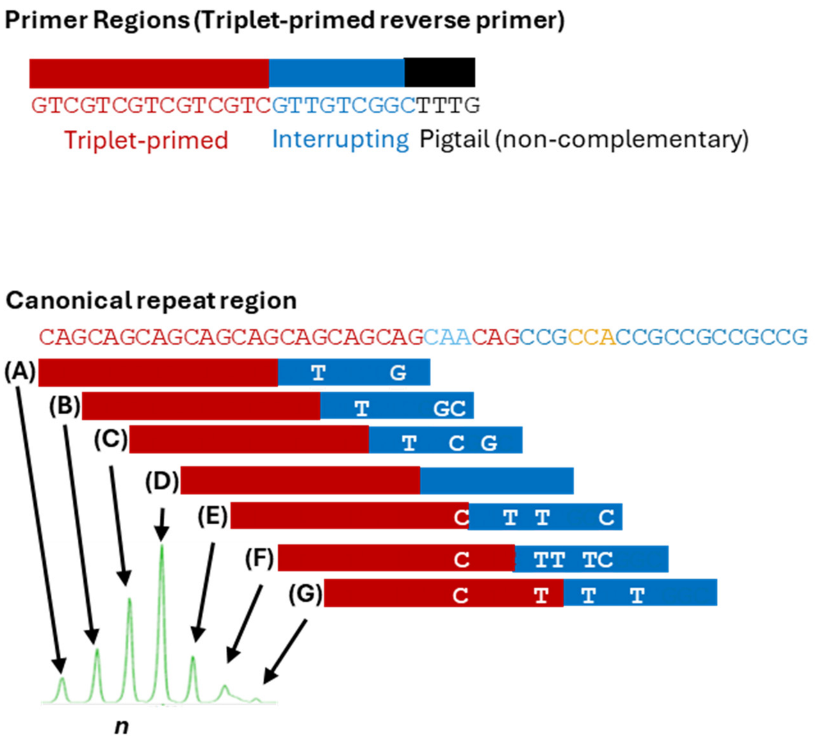


| Primer binding site | Peak Position | Number of mismatches | |
|---|---|---|---|
| | | Triplet-primed region (mismatches less tolerated) | Interrupting region (mismatches tolerated) |
| A | n-3 | 0 | 2 |
| B | n-2 | 0 | 3 |
| C | n-1 | 0 | 3 |
| D | n | 0 | 0 |
| E | n+1 | 1 | 3 |
| F | n+2 | 1 | 4 |
| G | n+3 | 2 | 2 |

*Supplementary 4: Triplet-primed reverse primer binding to canonical repeat region*

Diagram of primer binding to generate peaks seen in canonical allele trace. Triplet-primed region of the primer (red) is less tolerant of mismatches than the interrupting region (blue). Number of mismatches at each binding position is indicated in table form.

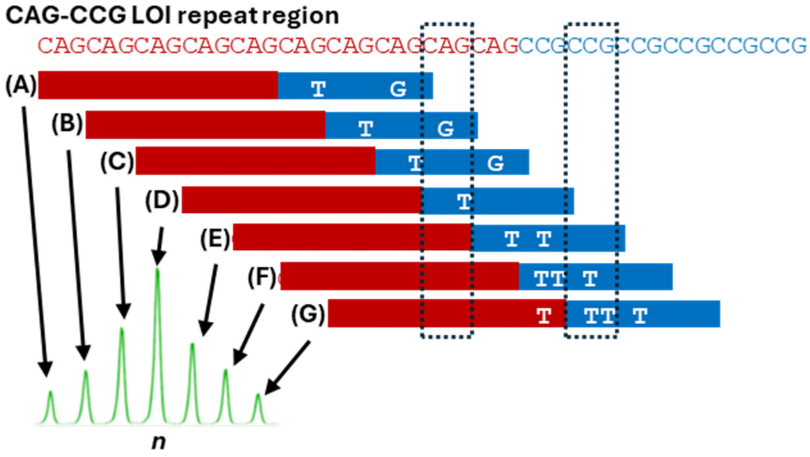


| Primer binding site | Peak Position | Number of mismatches | | Binding relative to canonical at same *n* position |
|---|---|---|---|---|
| | | Triplet-primed region (mismatches less tolerated) | Interrupting region (mismatches tolerated) | |
| A | n-3 | 0 | 2 | |
| B | n-2 | 0 | 2 | |
| C | n-1 | 0 | 2 | |
| D | n | 0 | 2 | -- |
| E | n+1 | 0 | 3 | + |
| F | n+2 | 0 | 3 | ++ |
| G | n+3 | 1 | 3 | = |

*Supplementary 5: Triplet-primed reverse primer binding to CAG-CCG LOI repeat region*

Diagram of primer binding to generate peaks seen in CAG-CCG LOI allele trace. Triplet-primed region of the primer (red) is less tolerant of mismatches than the interrupting region (blue). Number of mismatches at each binding position is indicated in table form, with a relative comparison to the number of mismatches at the same peak position for the canonical allele.

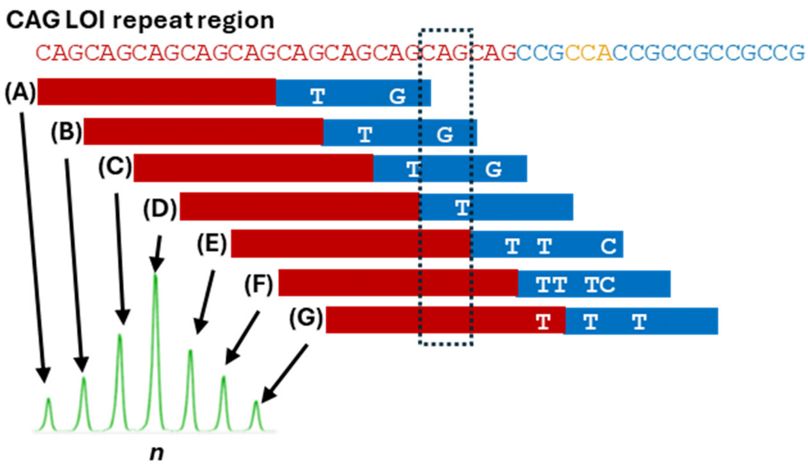


| Primer binding site | Peak Position | Number of mismatches | | Binding relative to canonical at same *n* position |
|---|---|---|---|---|
| | | Triplet-primed region (mismatches less tolerated) | Interrupting region (mismatches tolerated) | |
| A | n-3 | 0 | 2 | |
| B | n-2 | 0 | 2 | |
| C | n-1 | 0 | 2 | |
| D | n | 0 | 1 | - |
| E | n+1 | 0 | 3 | + |
| F | n+2 | 0 | 4 | + |
| G | n+3 | 1 | 2 | + |

*Supplementary 6: Triplet-primed reverse primer binding to CAG LOI repeat region*

Diagram of primer binding to generate peaks seen in CAG LOI allele trace. Triplet-primed region of the primer (red) is less tolerant of mismatches than the interrupting region (blue). Number of mismatches at each binding position is indicated in table form, with a relative comparison to the number of mismatches at the same peak position for the canonical allele.

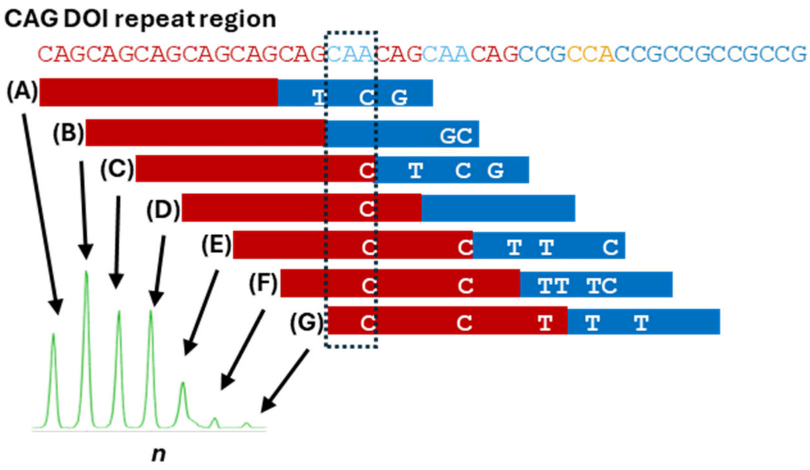


| Primer binding site | Peak Position | Number of mismatches | | Binding relative to canonical at same *n* position |
|---|---|---|---|---|
| | | Triplet-primed region (mismatches less tolerated) | Interrupting region (mismatches tolerated) | |
| A | n-1 | 0 | 3 | |
| B | n | 0 | 2 | -- |
| C | n+1 | 1 | 3 | = |
| D | n+2 | 1 | 0 | ++++ |
| E | n+3 | 2 | 3 | - |
| F | n+4 | 2 | 4 | |
| G | n+5 | 3 | 2 | |

*Supplementary 7: Triplet-primed reverse primer binding to CAG DOI repeat region*

Diagram of primer binding to generate peaks seen in CAG DOI allele trace. Triplet-primed region of the primer (red) is less tolerant of mismatches than the interrupting region (blue). Number of mismatches at each binding position is indicated in table form, with a relative comparison to the number of mismatches at the same peak position for the canonical allele.

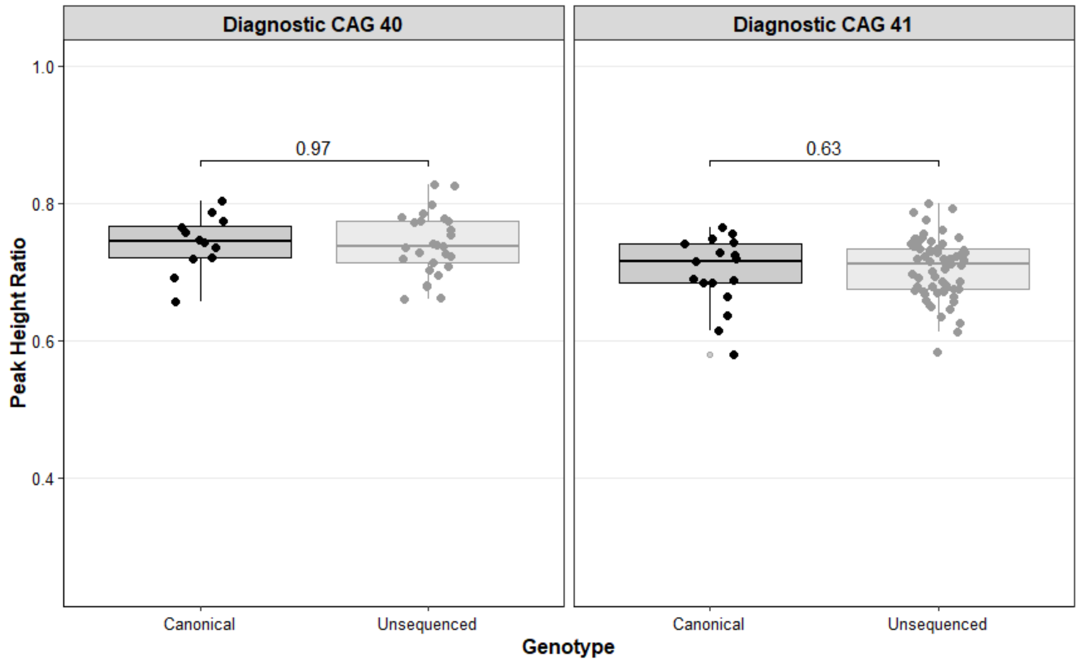


*Supplementary 8: Peak height ratio comparison of known- and presumed-canonical alleles*

Peak height ratio (PHR) values of sequenced canonical, and unsequenced presumed-canonical alleles with diagnostic CAG repeat lengths 40 and 41. P-values indicate results of T tests.

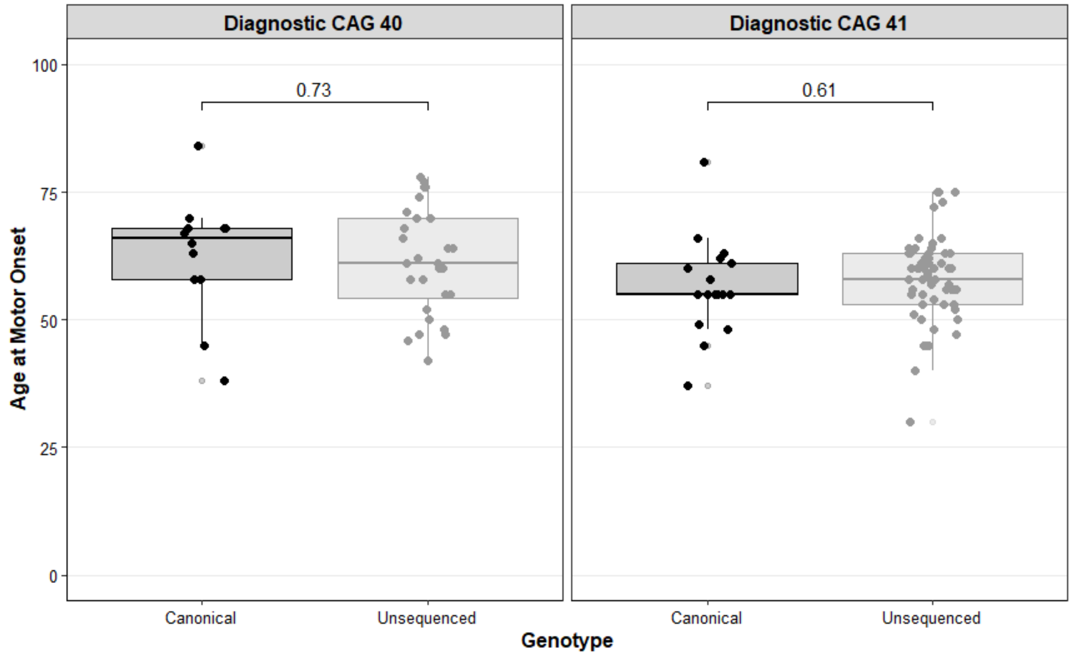


*Supplementary 9: Motor onset age comparison of known- and presumed-canonical alleles*

Age at motor onset of sequenced canonical, and unsequenced presumed-canonical alleles with diagnostic CAG repeat lengths 40 and 41. P-values indicate results of T tests.
